# H-polarized natural modes of microsize graphene strip grating in gain-material layer: regularizing Galerkin technique and lasing threshold conditions

**DOI:** 10.1098/rsta.2024.0350

**Published:** 2025-08-14

**Authors:** Tatiana L. Zinenko, Jiri Ctyroky, Oleksandr I. Nosych

**Affiliations:** ^1^ O Ya Usikov Institute for Radiophysics and Electronics National Academy of Sciences of Ukraine, Kharkiv, Ukraine; ^2^ Institute of Photonics and Electronics Czech Academy of Sciences, Prague, Czech Republic

**Keywords:** graphene strip grating, microsize laser, lasing eigenvalue problem, integral equation, Galerkin moment method discretization, localized surface plasmon

## Abstract

We study the threshold conditions for the natural modes of the microsize plasmonic laser shaped as an infinite flat graphene strip grating symmetrically embedded into the gain-material layer, in the H-polarization case. For this purpose, we solve the lasing eigenvalue problem (LEP), which is a classical source-free electromagnetic field boundary-value problem, adapted to the presence of the active region by the corresponding sign of the imaginary part of the refractive index. In such a way we look for the eigenpairs, i.e. the stimulated emission real-valued frequency and the threshold gain index, specific to each mode. We transform LEP to a hypersingular integral equation for the on-strip current density and discretize it by the regularizing Galerkin technique. This procedure leads to a determinantal equation with guaranteed convergence to the exact LEP eigenpairs and controlled accuracy of their computation. The numerical analysis allows us to study the threshold conditions for various lasing modes of the microsize laser, identify them and trace their change when varying the parameters of the lasing structure.

This article is part of the theme issue ‘Analytically grounded full-wave methods for advances in computational electromagnetics’.

## 1. Introduction

The advancement of lasers that use plasmonic effects signifies an encouraging direction in contemporary photonics [1]. A key breakthrough in miniaturizing the lasers was the introduction of noble metal-based nanoparticles as open cavities that function on the principles of plasmonics. This has led to the experimental demonstration of the tiniest plasmonic laser, which is based on colloidal gold nanospheres coated with dye-doped shells [2].

A common method for studying the natural modes of laser cavities used to be the classical complex-frequency eigenvalue problem based on the theory of source-free time-harmonic electromagnetic field for a passive open cavity. However, this approach is not entirely sufficient, as it overlooks the presence of the active region and does not account for the existence of the gain in this region [3]. As a consequence, the natural-mode frequency, *ω* (assuming the time dependence $e^{+j\omega t}$, for definiteness), can only be complex with a positive imaginary part that means the decay of the field in time that is not adequate for the lasing as a stationary field emission. In contrast, the LEP approach fully takes into account the size, shape and location of the active region [4–9]. Analysing the natural modes with the aid of this approach, we suppose that the refractive index of the gain material layer is *ν* = *α + iγ*, where *α* is the known value and *γ >* 0 is the unknown material gain provided by the pumping. This modification allows every natural-mode frequency to obtain a purely real value, thus making its field stationary in time. It suggests considering the LEP eigenvalues as ordered pairs, (*f_m_, γ_m_*), of the mode-specific real-valued eigenfrequencies, *f_m_* , and the corresponding threshold values of material gain in the active region, *γ_m_* [10–13], where *m* refers to the mode index. Importantly, the LEP approach is fully applicable to the analysis of the threshold conditions of the plasmonic lasers, i.e. those open-cavity configurations, which contain the noble-metal elements able to support the localized plasmon natural modes. Such full-wave electromagnetic analysis has been performed in [5,9] for the modes of silver nanostrip and nanotube, respectively, with circular quantum wires as active regions.

The appearance of graphene opens new possibilities in plasmonics since graphene monolayer is a material capable of guiding the surface plasmon polariton (SPP) natural wave, which is a wavelike collective oscillation of delocalized electrons, in the infrared- and THz-frequency ranges [14,15]. By patterning into strips and other shapes, graphene can form open resonators with the natural modes which are Fabry–Perot standing waves produced by the SPP natural wave of a graphene monolayer [16,17].

What makes graphene particularly appealing for applications is its conductivity, which can be dynamically controlled through the application of electrostatic bias. Currently, the most widely adopted quantum physical model of graphene conductivity is the Kubo model [14,15]. It demonstrates that the conductivity *σ* (*f*, *μ*_c_, *τ*, *T*) depends on chemical potential *μ*_c_, frequency *f*, electron relaxation time *τ* and temperature *T*. It involves two terms: intraband conductivity and interband conductivity. In the THz-frequency range, the intraband one yields the dominant contribution—see appendix A.

The ability to shape graphene into various forms, its excellent electronic, optical and mechanical properties as well as the dynamic control of its conductivity allows to develop novel photonic and plasmonic devices particularly in the THz and infrared ranges, including efficient antennas [18], electronically tuneable phase shifters [19], tuneable frequency selective surfaces [20] and plasmonic biosensors [21,22].

Patterned graphene shapes, including graphene-covered or graphene-strip-loaded circular quantum wires [4,23], are being evaluated as plasmonic micro-/nano-lasers. Graphene strip grating lasers are also attracting the attention of researchers [24]. In the full-wave electromagnetic modelling of graphene, the authors follow [14,15] and use two-side boundary conditions for a zero-thickness resistive surface [25,26].

In this paper, we consider, using the LEP, a microsize one-periodic graphene strip grating embedded into the gain material layer. In contrast to the noble-metal particle array-based nanolasers, including the silver strip grating on the gain substrate [27], the tunability of graphene promises obtaining the dynamically tuneable frequencies and thresholds of stimulated emission. As a numerical tool, we use a regularization technique which is a projection of the relevant hypersingular integral equation (HSIE) on a set of orthogonal polynomials that are the eigenfunctions of the singular part of the HSIE operator. This projection has been applied and validated in the analysis of the time-harmonic wave scattering from the same grating as in [16,17]. It leads to a well-conditioned determinantal equation for the LEP eigenpairs that entails mathematically guaranteed convergence of approximate eigenvalues to the exact ones if the matrix truncation order increases [28,29].

## 2. Theory

**Figure 1.**
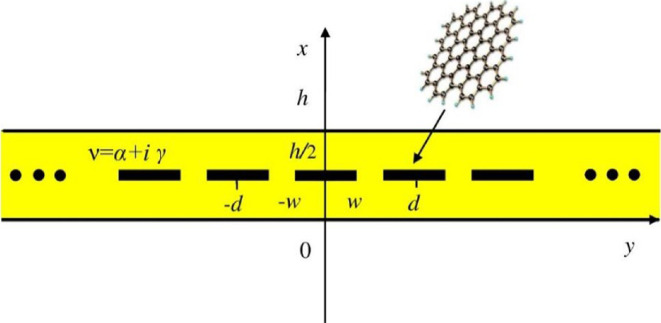
Cross-section of infinite flat graphene strip grating, symmetrically embedded into gain-material layer.

A cross-sectional view of the analysed two-dimensional LEP geometry is illustrated in figure 1. The flat zero-thickness strips are embedded into a gain-material layer with the complex refractive index *ν* = *α + iγ*, where *α* > 0 is a known value, *γ* > 0 is unknown threshold gain index, and the thickness is *h*. The layer is assumed non-magnetic, thus its complex relative permittivity is *ε_r_* = *ν*^2^. The grating is located in the middle plane of the layer at *x = h*/2; its period along the *y*-axis is *d* and the strip width is 2 w. The host medium is free space.

We will consider only the *H*-polarized time-harmonic electromagnetic field case as it is more important for applications owing to the existence, in contrast to the *E*-case, of the surface plasmon modes on graphene strips.

Thus, the LEP is formulated as a source-free electromagnetic boundary-value problem for the magnetic field function *H_z_*, which must satisfy

— Two-dimensional Helmholtz equation off the boundaries with coefficients $\varepsilon_{r}k^{2}$ in the layer and $k^{2}$ out of the layer.
— The resistive-sheet boundary conditions at the periodic sequence of graphene strip contours,

(2.1) $$\left\{ \begin{array}{l} \begin{array}{l} \left[{\vec{E}}_{T}^{+}\left(x,y\right)+{\vec{E}}_{T}^{-}\left(x,y\right)\right]=2\text{​}\text{​}\;Z\;\zeta_{0}\;\vec{x}\times \left[{\vec{H}}_{T}^{+}\left(x,y\right)-{\vec{H}}_{T}^{-}\left(x,y\right)\right],\\ {\vec{E}}_{T}^{+}\left(x,y\right)={\vec{E}}_{T}^{-}\left(x,y\right),\;\;\;x=h/2,\;\;|y-qd|<w,\;\;q=0,\pm 1,\;\pm 2...., \end{array} \end{array}\right.$$

where ζ_0_ is the free space impedance, *Z* is the normalized surface impedance of graphene (see appendix A), ± indicates the limiting values at x→h/2±0, *T* means tangential, and $\vec{x}$ is the unit vector.

— Continuity of the tangential field components across the material boundaries at *x* = 0 and *x = h*,

(2.2) $${\vec{E}}_{T}^{+}\left(x,y\right)={\vec{E}}_{T}^{-}\left(x,y\right),\;{\vec{H}}_{T}^{+}\left(x,y\right)={\vec{H}}_{T}^{-}\left(x,y\right).\;$$

— Radiation condition at |*x*| → ∞ that is consistent with the Principle of Radiation: the field must behave as the outgoing wave. Mathematically, the field must be expandable in terms of the Floquet series (2.5), where for every index *q*, either $Re\alpha_{0q}\geq 0$ or Imα0q≤0.
— Condition of the local power finiteness in any arbitrary domain *D* including the vicinity of strip edge,

(2.3) $$\int_{D}\left(|\nu^{2}\vec{E}{|}^{2}+Z_{0}^{2}|\vec{H}{|}^{2}\right)dxdy<\infty ,$$

that limits the field behaviour near the strip sharp edges. According to [30], in the case of the H-polarization, this entails the same edge behaviour of the electric current, ${\vec{J}}^{c}(y)=\vec{x}\times \left[{\vec{H}}_{T}(h/2+0,y)-{\vec{H}}_{T}(h/2-0,y)\right]$ as on the PEC strip, $O\left[{\left(y^{2}-w^{2}\right)}^{\;1/2}\right]$ if |*y*| → *w*.

These conditions are inherited from the time-harmonic wave scattering problem with real-valued *k*, where they guarantee the uniqueness of the scattered-field function provided that *k* is not the eigenvalue. When studying the threshold conditions for the natural modes of the grating, equipped with an active region, we look for the real-valued *k*; therefore, the same conditions are imposed.

From the grating periodicity and Floquet theorem, it follows that the field is a quasi-periodic function of *y*:


(2.4) $$H_{z}(x,y+d)=e^{j\beta_{0}d}H_{z}(x,y),$$

where $\beta_{0}$*d* is the field phase shift on single period (a.k.a. the Rayleigh parameter). Therefore, this function can be sought in the form of the Floquet–Rayleigh series of space harmonics,


(2.5) $$H_{z}\left(x,y\right)={\left(j\zeta_{0}\right)}^{\;-1}\sum_{q=-\infty }^{\infty }\rho_{H,q}\Phi_{H,q}\left(x\right)\;e^{-j\beta_{q}y}\;,$$

where $\rho_{H,q}$ are unknown Floquet harmonic amplitudes, $\beta_{q}=\beta_{0}+2q\pi /d$. Note that the Rayleigh parameter $\beta_{0}$ determines the propagation angle, $\varphi :\;\;\sin\varphi =\beta_{0}/k$ , of the zeroth Floquet harmonic with respect to the *x*-axis.

In the lasing threshold analysis, at least with flood pumping, there are no good reasons to consider that this parameter is non-zero. Therefore, we will further assume $\beta_{0}=0$, so that the zeroth harmonic radiates in the normal direction. However, this is not a critical restriction and arbitrary *β*_0_ does not spoil the technique used.

The functions $\Phi_{H,q}\left(x\right)$ satisfy the radiation condition and are expressed via the characteristic functions,


(2.6) $$\Phi_{H,q}(x)=\left\{ \begin{array}{ll} -\alpha_{1q}e^{j\alpha_{0q}x}/V_{H,q}^{e}(h/2), & x<0\\ -V_{H,q}^{o}(x)/V_{H,q}^{e}(h/2), & 0<x<h/2\\ V_{H,q}^{o}(h-x)/V_{H,q}^{e}(h/2), & h/2<x<h\\ \alpha_{1q}e^{-j\alpha_{0q}(x-h)}/V_{H,q}^{e}(h/2), & h<x \end{array}\right.$$

(2.7) $$V_{H,q}^{e}\left(x\right)=\alpha_{1q}sin\left(\alpha_{1q}x\right)-j\varepsilon_{r}\alpha_{0q}cos\left(\alpha_{1q}x\right),\;V_{H,q}^{o}\left(x\right)=\alpha_{1q}cos\left(\alpha_{1q}x\right)+j\varepsilon_{r}\alpha_{0q}sin\left(\alpha_{1q}x\right)$$

with transverse wavenumbers $\alpha_{1q}={\left(\varepsilon_{r}k^{2}-\beta_{q}^{2}\right)}^{1/2}$ inside and $\alpha_{0q}={\left(k^{2}-\beta_{q}^{2}\right)}^{1/2}$outside the layer. Note that the wavenumbers $k=\pm \beta_{q}$ (however, not $k=\pm \beta_{q}/\nu$) are the field (2.5) branch-points; they are known as the Rayleigh Anomalies (RAs).

The Fourier transform of the surface current density, extended by zero to the slot domain, allows us to obtain the integral representation of the *q*th Floquet harmonic amplitude, $\rho_{H,q}^{,}=-\zeta_{0}/2d\int_{-w}^{w}{\left(Y_{H,q}^{,}\right)}^{-1}J_{y}^{c}\left(y^{\prime }\right)\times e^{j\beta_{q}y^{\prime }}dy^{\prime },$ where $Y_{H,q}^{,}=V_{H,q}^{o}(h/2)/jV_{H,q}^{e}(h/2)$. On substituting this expression and (2.5) to the resistive boundary conditions (2.1), we obtain the integral equation of the second kind for the surface current, $F_{H}(t)=\zeta_{0}\;J_{y}^{c}(y^{\prime })\;e^{j\beta_{0}y^{\prime }}$$\left(y=w\;s,\;\;y^{\prime }=w\;t\right)$,


(2.8) $$Z\;F_{H}\left(s\right)\;+\int_{-1}^{1}K_{H}\left(s,t\right)\;F_{H}\left(t\right)\;dt=0\;$$

with the kernel function


(2.9) $$K_{H}^{,}\left(s,t\right)=\frac{j\Delta }{2\varepsilon_{r}kd}\sum_{q=-\infty ,even}^{\infty }\chi_{H,q}^{,}\Gamma_{H,q/2}^{,}\;e^{jq\left(t-s\right)\Delta },\;$$

where $\Delta =\pi w/d,\;\;\chi_{H,q}=-j\alpha_{1\;(q/2)}d/\pi$ if *q* is even or zero if *q* is odd, $\Gamma_{H,q}^{,}=1/Y_{H,q}^{,}$.

It is important to emphasize that, in the case of the *H*-polarization, the kernel function (2.9) is the expansion of the second derivative of the periodic Green’s function of the two-dimensional Helmholtz equation in terms of the Floquet harmonics, therefore, it is a hypersingular function as *s* → *t* and (2.8) is an HSIE. This becomes evident on using the asymptotic behaviour of $\Gamma_{H,\;q/2}∼1$ at | q |→∞ and transforming (2.9) as follows:


(2.10) $$K_{H}\left(s,t\right)=\frac{j}{2\varepsilon_{r}kd\Delta }\left[\frac{1}{{\left(t-s\right)}^{2}}\;\;+\;\Delta^{2}\sum_{q=-\infty }^{\infty }{\overset{˜}{\chi }}_{{\;}_{H,q}}e^{jq\left(t-s\right)\Delta }\right]$$

(2.11) $${\tilde{\chi }}_{H,q}=2\sum_{\nu =1}^{\infty }{\tilde{B}}_{H,\nu }\xi_{\nu q}+\left\{ \begin{array}{ll} \chi_{H,0}(Y_{H,0}{)}^{-1}+\frac{1}{3}, & q=0\\ \chi_{H,q}(Y_{H,q/2}{)}^{-1}+|q| & q\neq 0,\;even,\\ 0, & q\neq 0,\;odd \end{array}\right.$$

(2.12) $$\xi_{\nu 0}=\pi^{2\nu }/\left(2\nu +1\right)\;if\;q=0\;and\;\xi_{\nu q}=\left(2\nu \right)!\sum_{\mu =0}^{\nu -1}{\left(-1\right)}^{q+\mu }\pi^{2\left(\nu -\mu -1\right)}/\left(2\nu -2\mu -1\right)!q^{2\mu +2}\;if\;q\neq 0.$$

${\tilde{B}}_{H,\nu }=2^{2\nu }B_{\nu +1}{\left[\left(2\nu )!(\nu +1\right)\right]}^{\;-1}$ and $B_{\nu +1}$ are the Bernoulli numbers.

As is well known, discretization of such HSIE via the moment-method with the local basis functions does not lead to stable and convergent numerical code and should be replaced with more mathematically advanced techniques. Therefore, we discretize (2.8) using the technique based on the *regularizing Galerkin moment method* (MAR-Galerkin) [16,17,28,29]. Here, we need a set of orthogonal eigenfunctions of the extracted hypersingular part of the integral operator to apply as basis and testing functions in the projection method. This procedure is known to lead to an infinite matrix-operator equation of the Fredholm second kind. The weighted Chebyshev polynomials of the second kind form such a set for the canonical hypersingular operator represented by the first term in (2.10)—see (A 4) in appendix A; they also have the same behaviour at the strip edge as the unknown current function. Thus, we expand this function as


(2.13) $$F_{H}^{,}\left(t\right)=\sqrt{1-t^{2}}\sum_{n=1}^{\infty }f_{H,n}U_{n-1}\left(t\right).$$

Substituting the expansion (2.13) into HSIE (2.8), multiplying both parts of the equation with $\sqrt{1-s^{2}}U_{m-1}(s)$, and integrating in *s* from −1 to 1, we obtain the following infinite-matrix operator equation:


(2.14) $$\sum_{n=1}^{\infty }\left(\kappa_{H,mn}+Z{\overset{˜}{\kappa }}_{H,mn}\right)\;f_{H,n}=0,\;\;m=1,\;2,..,\infty .$$

Here, the matrix elements are represented by the following expressions:


(2.15) $$\kappa_{H,mn}^{,}=\int_{-1}^{1}\int_{-1}^{1}K_{H}^{,}\left(s,t\right)\;U_{m-1}\left(s\right)\;U_{n-1}\left(t\right)\;\sqrt{1-s^{2}}\;\sqrt{1-t^{2}}\;ds\;\;dt$$

(2.16) $${\overset{˜}{\kappa }}_{H,mn}=\int_{-1}^{1}U_{m-1}\left(s\right)U_{n-1}\left(s\right)\;\left(1-s^{2}\right)\;ds=\left\{ \begin{array}{l} \frac{1}{1-{\left(m-n\right)}^{2}}-\frac{1}{1-{\left(m+n\right)}^{2}}\;\left(m+n:\;even\right)\\ 0\;\;\;\;\;\;\;\;\;\text{​}\;\;\;\;\;\;\;\left(m+n:\;odd\right). \end{array}\right.$$

Since the weighted Chebyshev polynomials are orthogonal eigenfunctions of the hypersingular integral operator (see (A 4) and (A 5) in appendix A), the result of integration contains the Kronecker symbol *δ_mn_*.


(2.17) $$\kappa_{H,mn}^{,}=\frac{\pi }{2jkw\varepsilon_{r}}\left[\frac{n}{2}\delta_{mn}-\frac{\Delta^{2}}{4}{\overset{˜}{\chi }}_{H,0}\delta_{m1}\delta_{n1}-m\;n\;j^{n-m}\sum_{q=-\infty \left(q\neq 0\right)}^{\infty }\frac{{\overset{˜}{\chi }}_{H,q}}{q^{2}}J_{m}\left(q\Delta \right)\;J_{n}\left(q\Delta \right)\right],$$

where $J_{m}(q\Delta )$ is the Bessel function. Then, (2.14) turns to


(2.18) $$\sum_{n=1}^{\infty }\left(\delta_{mn}+A_{H,mn}\right)\;f_{H,n}=0,\;\;m=1,\;2,\;3,....$$

(2.19) $$\begin{array}{rl} A_{H,mn}=\; & 4\;j\;kw\varepsilon_{r}{\left(m\;\pi \right)}^{\;-1}Z{\overset{˜}{\kappa }}_{H,mn}-\Delta^{2}{\left(2m\right)}^{\;-1}{\overset{˜}{\chi }}_{H,0}\delta_{m1}\delta_{n1}\\ & -2\;n\;j^{n-m}\sum_{q=-\infty ,\neq 0}^{\infty }q^{\;-2}{\overset{˜}{\chi }}_{H,q}J_{m}\left(q\Delta \right)J_{n}\left(q\Delta \right). \end{array}$$

As the estimation $\sum_{n,m=1}^{+\infty }|A_{H,mn}{|}^{2}<\infty$ holds true, then (2.18) is the Fredholm second-kind matrix equation [28,29]. Finally, the search for the LEP eigenvalue pairs (*f, γ*) reduces to solving the determinantal equation,


(2.20) $$\det{\left[\delta_{mn}+A_{H,mn}^{,}(f,\gamma )\right]}_{т=1}^{\infty }=0,$$

and the regularized nature of (2.18) ensures the results converge when (2.20) is truncated to a finite order.

Besides the eigenvalues, the eigenvector of the current expansion coefficients, $f_{H,n}$, should also be computed in order to determine the natural mode Floquet harmonic amplitudes, $\rho_{Н,q}$,


(2.21) $$\rho_{H,q}=\left\{ \begin{array}{l} -\Delta \;f_{H,1}{\left(4Y_{H,0}\right)}^{-1},\;\;\;\;q=0\\ {\left(4qY_{H,q}\right)}^{-1}\sum_{n=1}^{\infty }f_{H,n}n\text{​}j^{n+1}J_{n}\left(2q\Delta \right),\;\;q\neq 0. \end{array}\right.$$

Then, we can restore the mode magnetic field pattern according to (2.5).

## 3. Numerical results

**Figure 2.**
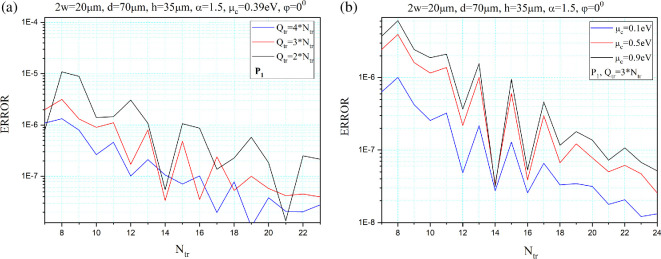
Computational error in the eigenpair of the plasmon mode *P*_1_ as a function of the matrix truncation number for three values of the *Q*_tr_/*N*_tr_ (a) and three values of the chemical potential *μ_c_* (b). *τ* = 1 ps, *T* = 300 K.

In figure 2a, one can see the computational error in the eigenpair of the first plasmon mode *P*_1_ as a function of *N*_tr_ for three values of the *Q*_tr_/*N*_tr_ ratio, where *Q*_tr_ is the truncation number of the Floquet–Rayleigh series of space harmonics ( 2.5) and *N*_tr_ is the truncation number of the surface current density series expansion by Chebyshev polynomials of the second kind as the basis functions in Galerkin’s procedure.

As follows from figure 2a, our algorithm shows a rapid decrement of the relative truncation error, $Er=\sqrt{{\left(\delta \text{f}\right)}^{2}+{\left(\delta \gamma \right)}^{2}},$ where $\delta f=|f_{Ntr}-f_{Ntr+1}||f_{Ntr+1}{|}^{\;-1},\;\delta \gamma =|\gamma_{Ntr}-\gamma_{Ntr+1}||\gamma_{Ntr+1}{|}^{\;-1}$, although with oscillations, as the truncation number *N*_tr_ increases. Besides, it should be noted that the convergence rate increases with increasing truncation numbers ratio *Q*_tr_/*N*_tr_, as shown in figure 2a. Figure 2b shows the decay of the relative truncation error for three different chemical potential values *μ*_c_ and *Q*_tr_ = 3_Ntr_. As can be seen, the convergence rate is almost the same for all considered chemical potentials *μ_c_* and hence for the surface impedance *Z* values. This is a consequence of the fact that the singularity of HSIE and the regularization method do not depend on *Z* and coincide with the case of the PEC strip grating. Still, the error itself scales as |*Z*|.

**Figure 3.**
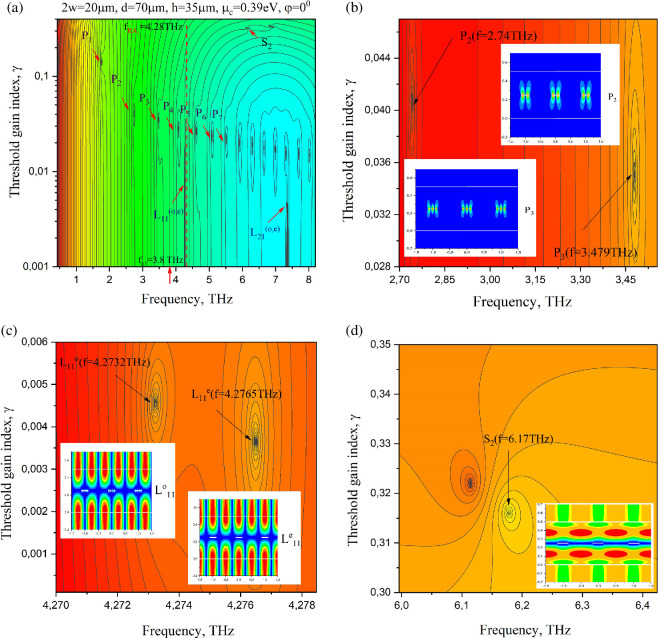
The colour map of the LEP determinant absolute value for the laser configuration from figure 1, on the plane (*f*,*γ*) (a); zoom around the plasmon modes *P*_2_ and *P*_3_ (b); zoom around the lattice modes, $L_{11}^{e}$ and $L_{11}^{o}$ (c); zoom around the slab mode $S_{2}$ (d). The insets on panels (b,c) and (d) are the near magnetic field patterns for the corresponding modes.

To accurately compute the values of frequency and threshold gain, we employ the modified hybrid Powell algorithm from the IMSL library. This iterative algorithm requires specific initial guess values to proceed efficiently. To obtain reliable initial guesses, we first build a map of the determinant absolute value as a function of the frequency *f* and threshold gain index *γ* in the analysed ‘window.’ Such a map displays local minima—see figure 3a, computed with *N*_tr_ = 20, *Q*_tr_ = 60 for 2*w* = 20 μm, *d* = 70 μm, *h* = 35 μm and *α* = 1.5. The initial guesses are taken from the minima where the determinant is close to zero and then refined through iterations.

Notably, some of the eigenpairs in figure 3a lie on a hyperbolic curve, *γ* = const *f* ^−1^—see appendix A. They correspond to the plasmon modes of the graphene strips [4]. Such identification is supported by their near magnetic field portraits, which display characteristic bright spots confined to the graphene strips—see *P*_2_ and *P*_3_ in figure 3b. These modes are formed due to the bouncing of the surface plasmon natural wave of graphene monolayer between the strip edges.

Besides the plasmon modes, one can see ultralow-threshold lattice modes labelled as $L_{11}^{\left(o.e\right)}$ and $L_{21}^{\left(o,e\right)}$. Their near-field patterns, shown in figure 3c, have an even number of bright field spots on the grating period. These are the modes of the grating-loaded dielectric slab as a periodic open resonator [10,13,16,17]. The frequencies of the lattice modes are defined primarily by the period of the grating, i.e. are close to the RAs. However, the red shift from RA is defined by the propagation constant of one of the guided natural waves of the bare dielectric slab as an open waveguide that scales with layer refractive index and thickness. Therefore, the first index of the lattice mode corresponds to the nearest RA, i.e. to the ±1st or the ±2nd one; see the red dashed line at 4.28 THz. The second index, here 1, corresponds to the index of the *TM*_1_ guided natural wave of the dielectric slab with cutoff frequency *f*_c_ = 3.8 THz indicated by the red arrow in figure 3a. The superscripts *e* and *o* denote the *y*-even and *y*-odd classes of the mode magnetic field symmetry.

Besides, one can see at *f* = 6.17 THz the high-threshold mode S_2_ of the active dielectric layer slightly perturbed by the presence of partially transparent and lossy graphene strips. Note that the lattice-mode thresholds are 2 orders lower than those of the slab modes, which have very high radiation losses.

**Figure 4.**
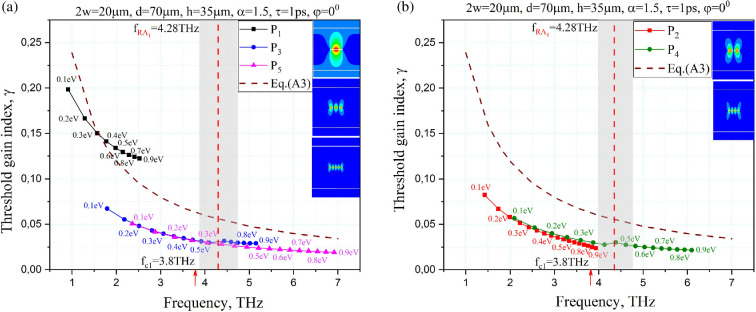
The trajectories of the LEP eigenvalues of the *y*-even plasmon modes *P*_1_, *P*_3_ and *P*_5_ (a) and *y*-odd modes *P*_2_ and *P*_4_ (b) on the plane (*f*, *γ*) as a function of the graphene chemical potential for the structure with the same parameters as in figure 3. The insets are the magnetic field patterns for these plasmon modes at $\mu_{c}=0.39\;eV$.

Further, we investigate only the plasmon-mode eigenvalues because they are well tuneable with the aid of the change of graphene chemical potential [14–17]. Figure 4 shows the trajectories of the LEP eigenpairs ($f_{m}^{p}$,$\gamma_{m}^{p}$) of the plasmon modes at the chemical potential varying from 0.1 to 0.9 eV with the step 0.1 eV.

Here, we use the same geometrical parameters of the structure as in figure 3, i.e. the strip width is 20 μm, the grating period is 70 μm, the gain layer thickness is 35 μm, the electron relaxation time is *τ* = 1 ps, and the temperature is 300 K. Presented are the trajectories of five plasmonic modes, P_1_*–P*_5_, with their magnetic near-field portraits. As can be seen, the plasmon-mode eigenpairs are tuneable with the aid of the graphene chemical potential within a factor of 3. The plasmon mode *P*_1_ has the largest threshold value over the whole range compared to the plasmon modes of the larger indices. By increasing the chemical potential, the mode eigenpairs move approximately along the mentioned hyperbola, shown by the dash-dotted curve in figure 4.

The grey vertical strip around RA_1_ indicates the area of hybridization of the $L_{11}^{\left(o,e\right)}$ lattice modes with the plasmon modes of the indices greater than 2, i.e. with *P*_3_, *P*_4_ and *P*_5_, which makes it difficult to achieve a single-mode operation regime for them in this frequency region. Similar hybridization takes place for *P*_1_ and *P*_2_ at chemical potential values larger than 1 eV. We leave the analysis of the mode hybridization to a separate publication as it needs a more in-depth study and additional space.

**Figure 5.**
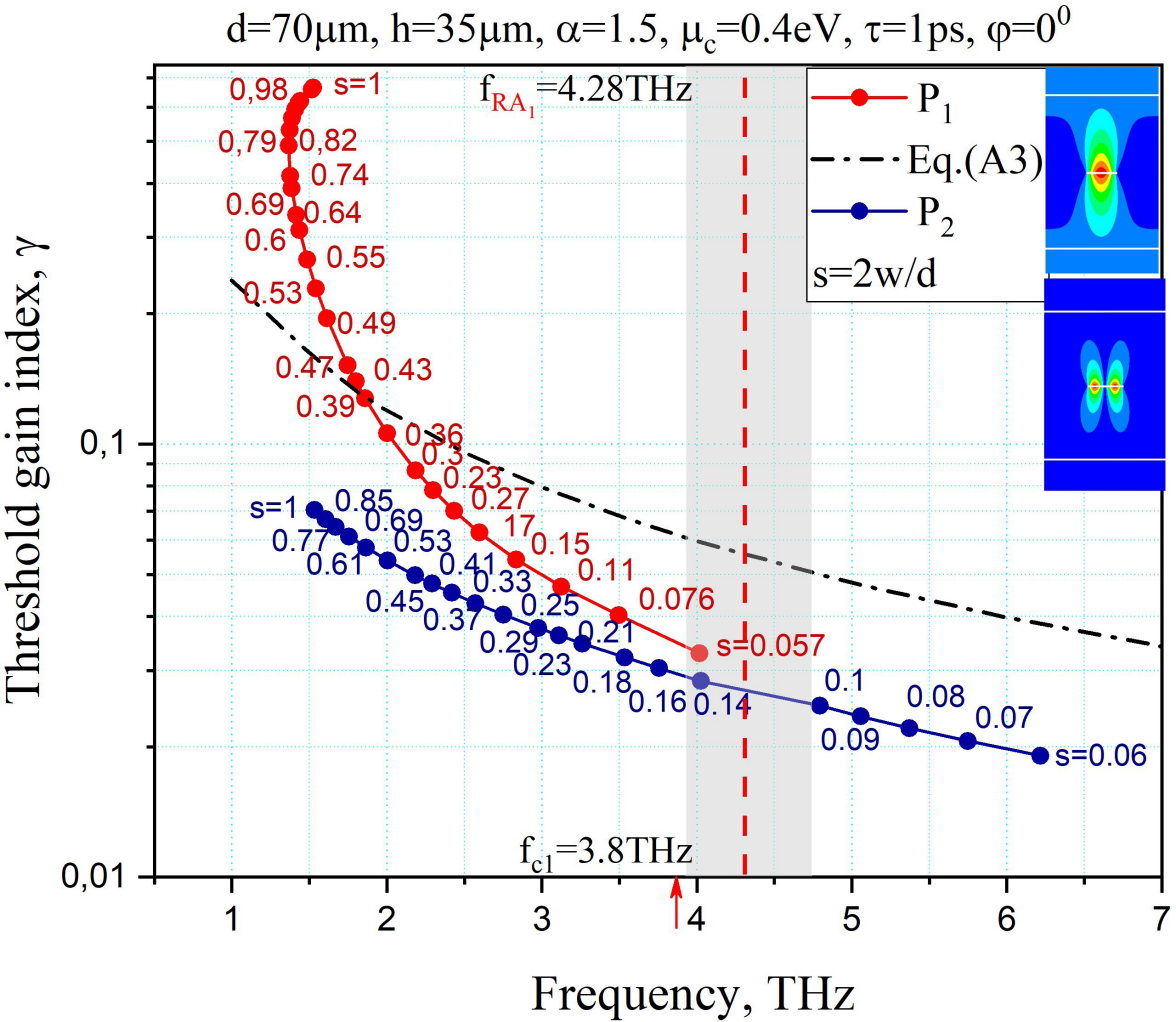
The trajectories of the LEP eigenvalues of the plasmon modes *P*_1_ and *P*_2_ on the plane (*f*, *γ*) as a function of the filling factor of the graphene strip grating. The structural parameters are the same as in figure 3.

In figure 5, the trajectories of the LEP eigenvalues of the plasmon modes *P*_1_ and *P*_2_ on the plane (*f*, *γ*) are studied under the variation of the grating filling factor, 2 *w*/*d*. Although they are both close to the mentioned hyperbola (dash-dotted curve), their behaviours are significantly different at the large filling factor values, 2 *w*/*d* ≈ 1.

Namely, if the grating is dense, then the *P*_1_ and *P*_2_ modes have comparable frequencies, however, *P*_1_ has an order higher threshold gain values than those of *P*_2_.

**Figure 6.**
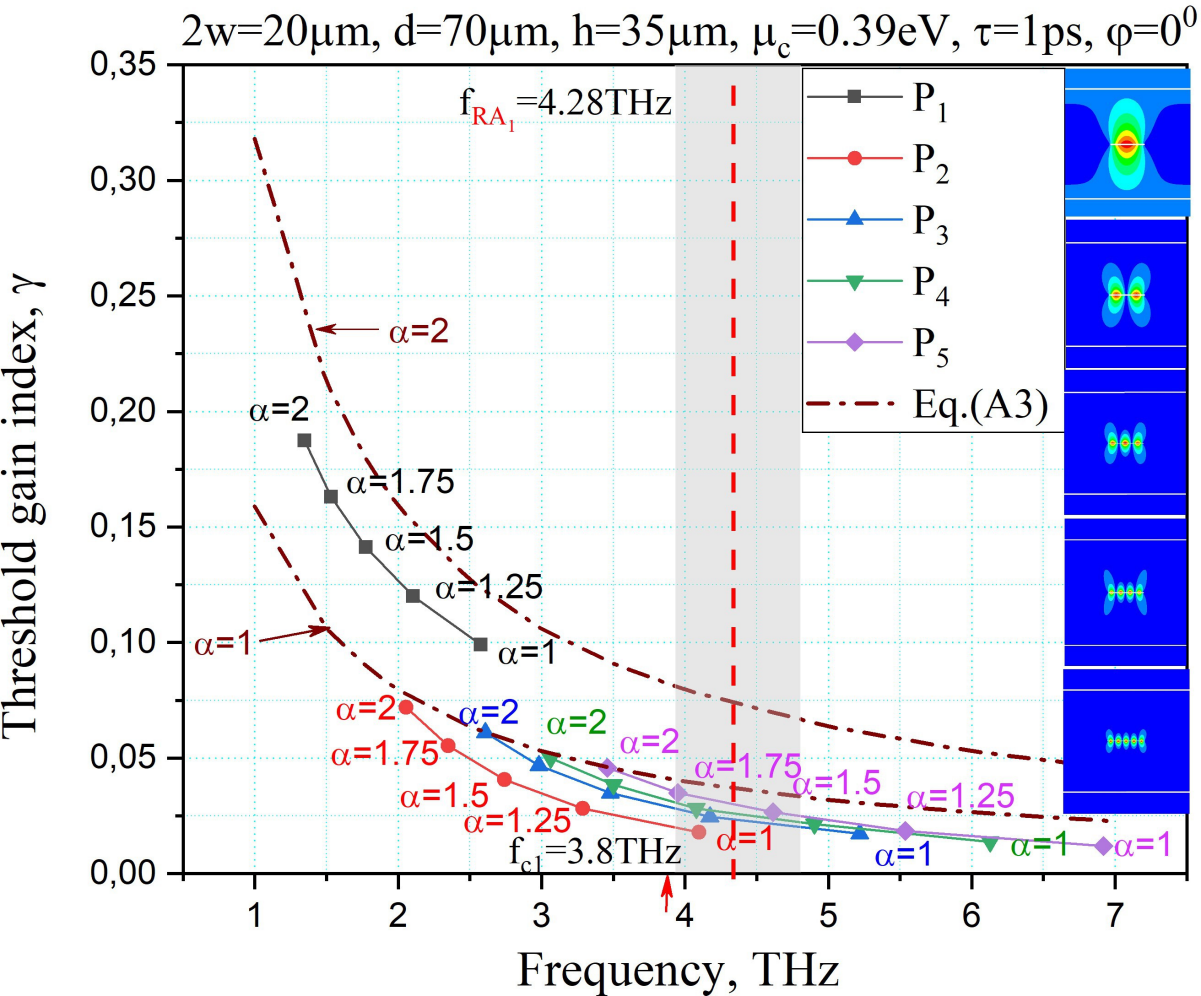
The trajectories of the eigenvalues of the plasmon modes on the plane (*f*, *γ*) as a function of the gain-material refractive index. The structural parameters are the same as in figure 3.

Figure 6 demonstrates the trajectories of LEP eigenvalues of the plasmon modes *P*_1_ to *P*_5_ on the plane (*f*, *γ*) when the real part of the gain-material layer refractive index, *α*, is changed between 1 and 2. Similar to figures 4 and 5, trajectories follow a hyperbola and the mode *P*_1_ has higher threshold values in comparison to the plasmon modes with higher indices. For comparison, the eigenpairs trajectory based on the analytical approximate formula (A 3)—see appendix A, at *α* = 1 and *α* = 2—are also presented as brown dash-dotted lines.

**Figure 7.**
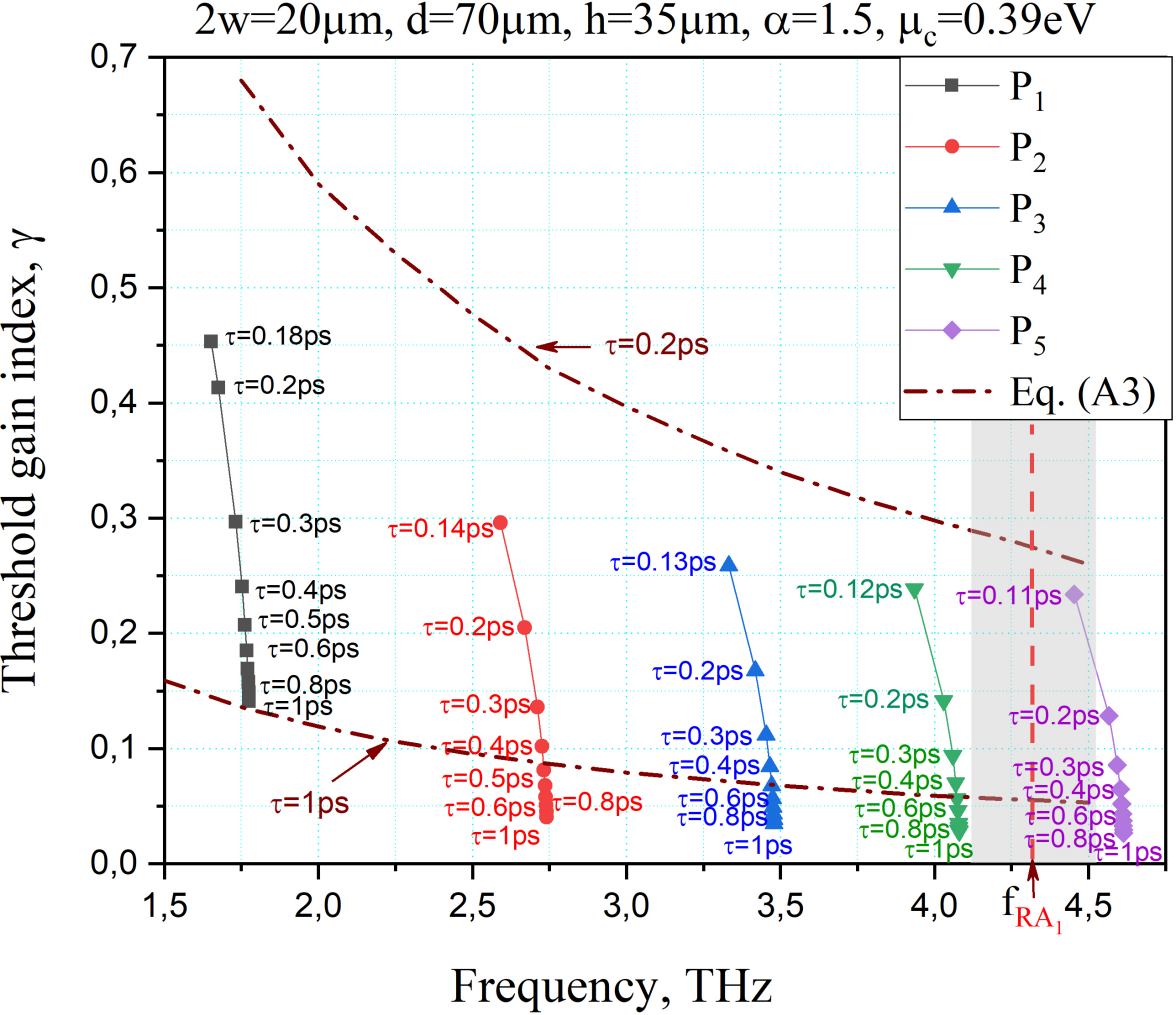
The trajectories of the LEP eigenvalues of the plasmon modes on the plane (*f*, *γ*) as a function of the graphene electron relaxation time. The structural parameters are the same as in figure 3.

Finally, figure 7 shows the trajectories of the LEP eigenvalues of the plasmon modes *P*_1_*–P*_5_ on the plane (*f*, *γ*) when the graphene relaxation time *τ* varies from 0.2 to 1 ps. For comparison, the curves of approximate relationship (A 3) for *τ* = 0.2 and *τ* = 1 eV are also shown as dash-dotted lines. The curves calculated using the full-wave determinant equation (2.20) demonstrate the same behaviour as for the graphene-wire laser embedded into a circular active region [23], i.e. the larger *τ*, the lower the thresholds of all plasmon modes.

## 4. Conclusions

We have used the accurate numerical technique developed earlier by us to study the wave-scattering problems, and to analyse the LEP for the H-polarized modes of a laser designed as the infinite graphene strip grating embedded into the layer of gain material. It is based on the hypersingular integral equation for the strip current, discretized by the MAR-Galerkin method, i.e. it uses the weighted Chebyshev polynomials of the second kind as full-wave expansion functions. Owing to the fact that these polynomials are the eigenfunctions of the hypersingular part of the full integral operator, the projection procedure leads to a Fredholm second-kind infinite matrix equation for the current expansion coefficients. The determinant of that equation provides the LEP eigenpairs of all the natural modes of the studied laser configuration. We have investigated the computational errors for the eigenpairs as a function of the matrix truncation number and the graphene chemical potential and have demonstrated the fast convergence of the solution.

If the grating and layer dimensions are in the microsize range, the natural-mode frequencies are in the THz-frequency range. Considering the behaviour of the eigenpairs on the colour map of the LEP determinant absolute value, we have identified various lasing modes of the microscale laser and determined their threshold conditions dynamics under the variation of various parameters such as graphene chemical potential, filling factor of the grating, gain-material layer refractive index and graphene electron relaxation time. As we have found, the lattice mode thresholds are the lowest ones, however, the best tunability with the aid of graphene chemical potential, within a factor of 3, is demonstrated by the plasmon modes. Under variation of the graphene chemical potential or the strip width, the plasmon modes change their frequencies and may enter the hybridization regime with the lattice modes. This phenomenon needs a deeper analysis, which we hope to present in a separate publication.

The real-life gratings have, of course, finite dimensions. To the question of possible deviations from the above-presented theory, caused by that circumstance, a partial answer can be found in the analysis of the plane-wave scattering from finite graphene strip gratings, suspended in the free space [31]. As visible from plots in fig. 11 of [31], the gratings of 50 or more strips, each 20 μm wide and placed at 70 μm intervals, demonstrate the same per-strip reflectance as infinite strip grating in the whole THz range, including all plasmon-mode resonances, except narrow vicinities of RAs. Today’s graphene strip grating sensors contain many hundreds and even thousands of strips [22], and hence their characteristics should be even better reproduced by the infinite-grating model. The same can be expected of the mode threshold conditions for the finite-grating laser configurations. Namely, the plasmon-mode and slab-mode thresholds should be rather insensitive to the number of strips, however, the lattice modes should demonstrate the thresholds, depending on this number until it reaches hundreds or thousands, depending on the configuration.

The zero-thickness Kubo model of graphene monolayer is another source of possible deviation from the real-life characteristics. Here, it can be noted that, in fact, the measurements show that graphene usually has a 2−4 nm thickness [22] which points to the presence of a stack of several monolayers. It is commonly considered that as long as the number of monolayers is small, less than 10, it should be added as a factor to the expressions of the complex conductivity (A 1) in order to obtain the conductivity of the stack.

We have performed our analysis assuming that the gain index, *γ*, is uniform, i.e. not dependent on the frequency, while in reality, it is usually characterized by a Lorentzian-like spectrum. However, it is easy to see that if the gain is uniform within a finite interval and vanishes off it, then all modes that have their frequencies in this interval keep their thresholds the same as above, while the thresholds of the other modes turn to infinity.

## Data Availability

All computations relating to the results presented in this paper can be readily reproduced by a reader by using the equations explicitly provided in the paper.

## Appendix A.

Today, the most widely recognized model of the electron conductivity of the graphene is the Kubo formalism. At the frequencies up to the X-rays, the graphene monolayer thickness can be safely assumed zero, and its 10-nm scale hexagonal fine structure can be neglected. Under these assumptions, graphene’s surface conductivity has two contributions, $\sigma =\sigma_{\text{intra}}+\sigma_{\text{inter}}$, which are the intraband and interband conductivities. Namely, the first of them is also known as Drude-like conductivity,

(A 1)
$$\sigma_{\text{intra}}=-\frac{j\Omega \zeta_{0}}{\omega -j\tau^{-1}},\;\Omega =\frac{q_{e}^{2}k_{B}T}{\pi \hbar^{2}\zeta_{0}}\left\{\frac{\mu_{c}}{k_{B}T}+2\ln\left[1+\exp\left(-\frac{\mu_{c}}{k_{B}T}\right)\right]\right\},$$

where $\zeta_{0}=\sqrt{\mu_{0}/\varepsilon_{0}}$ is the free space impedance. The second term, $\sigma_{\text{inter}}$, is expressed as integral of known functions [14,15]. The normalized surface impedance (or resistivity) of graphene is $Z(\omega )=\zeta_{0}^{\;-1}{\left(\sigma_{\text{intra}}+\sigma_{\text{inter}}\right)}^{\;-1}.$

The relative contribution of two terms to *Z* depends on the frequency and chemical potential [14,15,23], so that at $\mu_{c}=1\text{ eV}$, $\sigma_{\text{inter}}$ is less than 0.001 of $\sigma_{\text{intra}}$, in absolute value, if the frequency is below 60 THz. This allows to neglect the radiation losses and derive approximate expressions for the threshold characteristics of the plasmon natural modes of the flat graphene strip embedded into the gain medium [4]. Their emission frequencies, $f_{m}^{P}=k_{m}^{P}c/2\pi$, and the threshold values of gain are found as (*m* = 1,2,3,…),

(A 2)
$$f_{m}^{P}\approx \frac{1}{4\pi \alpha }{\left[\frac{\pi \left(m-0.25\right)c\;\Omega }{w}\right]}^{\;1/2},\;\;\gamma_{m}^{P}\approx \frac{2\alpha^{2}}{\tau }{\left[\frac{w}{\pi \left(m-0.25\right)c\;\Omega }\right]}^{1/2}.$$

From (2.19) it follows that the LEP eigenpairs satisfy the equation,

(A 3)
$$\gamma_{m}^{P}\cdot f_{m}^{P}\approx \alpha {\left(2\pi \tau \right)}^{-1},$$

which is valid provided that the plasmon modes ohmic losses in graphene dominate over the radiation losses.

∗∗∗

Orthogonality of the weighted Chebyshev polynomials of the second kind and their property as the eigenfunctions of the hypersingular part of the full integral operator are expressed as follows:

(A 4)
$$\int_{-1}^{1}\sqrt{1-s^{2}}U_{m-1}\left(s\right)\;U_{n-1}\left(s\right)ds=\pi \delta_{mn}/2\;\;,$$

(A 5)
$$\int_{-1}^{1}\frac{\sqrt{1-t^{2}}U_{n-1}\left(t\right)}{{\left(t-s\right)}^{\;2}}dt\;=-n\pi \;U_{n-1}\left(s\right)\;.$$
